# Integrated Care Models for Managing and Preventing Frailty: A Systematic Review for the European Joint Action on Frailty Prevention (ADVANTAGE JA)

**Published:** 2019-01-06

**Authors:** A Hendry, E Vanhecke, AM Carriazo, L López-Samaniego, JM Espinosa, D Sezgin, M O’Donovan, T Hammar, P Ferry, A Vella, OA Bacaicoa, M Braga, M Ciutan, A Velivasi, M Lamprini Koula, J Van der Heyden, A Liew, R O’Caoimh

**Affiliations:** 1 NHS Lanarkshire, United Kingdom; 2 Ministry of Health and Social Affairs, France; 3 Consejeria de Salud de la Junta de Andalucia, Sevilla, Spain; 4 Fundación Progreso y Salud, Consejería de Salud de la Junta de Andalucía, Sevilla, Spain; 5 Servicio Andaluz de Salud, Sevilla, Spain; 6 Clinical Sciences Institute, National University of Ireland, Galway, Galway City, Ireland; 7 National Institute for Health and Welfare, Finland, Helsinki, Finland; 8 Ministry for the Family and Social Solidarity, Malta; 9 Centro de Excelencia en Investigación en Cronicidad (KRONIKGUNE), Barakaldo, Spain; 10 Agenzia Nazionale per i Servizi Sanitari Regionali, Italy; 11 Scoala Nationala de Sanatate Publica, Management si Perfectionare in Domeniul Sanitar, Bucharest, Romania; 12 Society of Psychosocial Research and Intervention. Ioannina, Greece; 13 Sciensano, Brussels, Belgium

**Keywords:** Frailty, models of care, systematic review, integrated care

## Abstract

Frailty requires concerted integrated approaches to prevent functional decline. Although there is evidence that integrating care is effective for older people, there is insufficient data on outcomes from studies implementing integrated care to prevent and manage frailty. We systematically searched PubMed and Cochrane Library database for peer-reviewed medical literature on models of care for frailty, published from 2002 to 2017. We considered the effective and transferable components of the models of care and evidence of economic impact, where available. Information on European Union-funded projects or those registered with the European Innovation Partnership on Active and Healthy Ageing, and grey literature (including good practices) were also considered. We found 1,065 potential citations and 170 relevant abstracts. After excluding reports on specific diseases, processes or interventions and service models that did not report data, 42 full papers met the inclusion criteria. The evidence showed that few models of integrated care were specifically designed to prevent and tackle frailty in the community and at the interface between primary and secondary (hospital) care. Current evidence supports the case for a more holistic and salutogenic response to frailty, blending a chronic care approach with education, enablement and rehabilitation to optimise function, particularly at times of a sudden deterioration in health, or when transitioning between home, hospital or care home. In all care settings, these approaches should be supported by comprehensive assessment and multidimensional interventions tailored to modifiable physical, psychological, cognitive and social factors.

## I. INTRODUCTION

Frailty is a common [1] complex syndrome predisposing to negative health and social care outcomes but is multi-dimensional and difficult to define [2]. Although the terms are not synonymous [3], frailty, disability and multimorbidity are complementary concepts associated with clinical complexity, increased use of healthcare resources and higher costs [4]. Frailty shares many features of a chronic condition: a dynamic largely fixed syndrome [5] that may be prevented and is better managed in primary care through an interdisciplinary chronic disease management approach that anticipates and proactively manages episodes of deteriorating function [6]. Tackling frailty is recognised as a priority in the European Union (EU) [7] resulting in initiatives such as the European Innovation Partnership on Active and Healthy Ageing (EIPAHA) [8]. Interventions common to both multimorbidity and frailty include proactive assessment, care planning and review; coordination of care; targeted enablement and support for self-management; and behaviour change approaches that go beyond the scope of a traditional biomedical approach [9]. Integrated care has emerged as an effective way to improve outcomes for older people with chronic and complex care and support needs [10]. Many chronic care programmes aim to deliver integrated care through the building of continuous relationships with a primary care or social care professional, supported by coordinated care from an interdisciplinary team [11]. It is widely suggested that integrated care may be most effective when applied to an older population, but there is limited data to support this hypothesis [12].

The ADVANTAGE Joint Action (JA) is an EU funded project that aims to develop a common European Prevention of Frailty Approach [13]. Work Package 7 (WP7) aims to identify models of care to prevent or delay progression of frailty and enable people to live well with frailty. We explored the following questions: “What are the core concepts within models of integrated care?”, “What is the experience of implementing models of integrated care for frailty?”, “What are the outcomes from adopting integrated care for people who are frail?” and “What are the implications for future research and education on integrated care for frailty?”

## II. METHODOLOGY

### Eligibility criteria

A systematic search of peer-reviewed medical literature published from 2002 to 2017 was undertaken to identify articles assessing the impact of models of care to prevent or manage frailty. The Preferred Reporting Items for Systematic Reviews and Meta-Analyses (PRISMA) guidelines [14] were used.

### Information sources

The search was conducted using PubMed and Cochrane Library database by combining two key concepts: a frailty approach and models of care. For the grey literature review, the websites of relevant frailty, multi-morbidity or integrated care projects funded by the EU were reviewed. We also reviewed a compilation of 286 practices gathered by the EIPAHA Frailty Action Group and a compendium of peer – reviewed examples of excellent innovation in ageing from 32 EIPAHA Reference Sites.

### Search strategy

Search terms “frailty” or “frail” were applied without prejudice as to the specific definition. The specific query translation is included in Appendix 1.

WP7 partners were invited to submit ‘grey literature’ on models of care for frailty from their country. This could include examples of a good practice, defined as a practice *“that has been proven to work well and produce good results, and is therefore recommended as a model … a successful experience, which has been tested and validated, in the broad sense, which has been repeated and deserves to be shared so that a greater number of people can adopt it”*.

#### Study selection

**Fig. 1:**
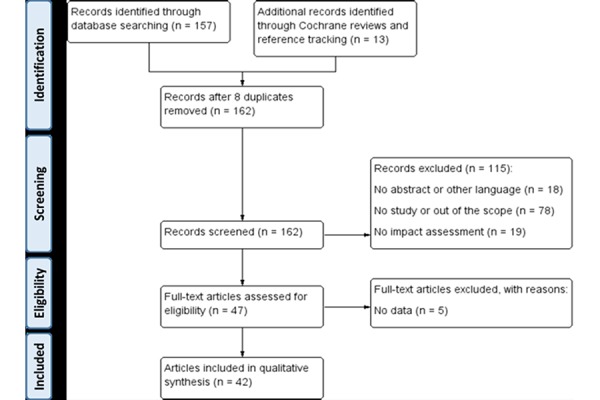
PRISMA flow diagram.

As a first search of titles and abstracts identified 1065 potential articles other databases were not searched. A more filtered review of titles identified 157 abstracts (42 from the first query and 115 from the second) of interest. Systematic reviews on Comprehensive Geriatric Assessment (CGA) and Intermediate care models were also analysed with reference tracking (Figure 1).

### Data collection process

After applying exclusion criteria (focused on a specific disease or intervention without considering service delivery, or lack of data on impact), 42 articles were analysed using a standard template.

### Synthesis of the results and additional analyses

A qualitative approach was followed for the synthesis of the results.

## III. RESULTS

The good practices submitted are presented in Appendix 2.

A systematic review of 18 comprehensive integrated care programmes for people with multimorbidity or frailty, [15] reported some evidence of improved health-related quality of life, function, and satisfaction with care but no reduction in health services utilisation or costs. All included innovations such as appointing case managers, establishing multi-professional teams, and implementing individualised care plans.

Comprehensive assessment, individualised care plans, and coordination of tailored interventions are the essence of both integrated care and of CGA: a highly evidenced approach that improves outcomes for frail older people in hospital [16]. This review considers the evidence for comprehensive assessment and integrated care approaches applied at key points in the frailty care pathway.

### Preventative education, enablement and care and support at home

Ryburn et al., [17] reviewed three non-randomised controlled trials of restorative home care (home support designed to enable recovery of independence). The intervention improved self-care, activities of daily living, mobility and morale, reduced falls and need for home care, increased the likelihood of remaining at home, and reduced visits to an emergency department. In a non-randomised, controlled study of 252 community-based older people and their caregivers, preventative interventions resulted in high levels of patient and caregiver satisfaction, reduced cognitive impairment and depression [18].

Markle-Reid et al., [19] reported on three single blind randomised studies of nurse led education on falls prevention, nutrition and self-management. The intervention group reported improved health related quality of life, reduced depression, enhanced perception of social support, significantly lower cost of prescription medications, but no difference in the cost of services.

A quasi-experimental study of integrated care reported greater caregiver support and satisfaction, reduced anxiety and caregiver burden and caregivers were more likely to continue to provide assistance at home [20].

### Comprehensive Assessment and Chronic Case Management in Primary Care

In a meta-analysis of 89 randomised controlled trials of comprehensive and complex community interventions, Beswick et al. [21] reported some evidence for improved physical function and a reduction in falls, hospital admissions and admissions to care homes. However, the greatest benefit was observed in the early studies raising questions about the applicability of the findings within the current model of primary care.

Béland et al. [22] analysed nine international examples of integrated primary care for frail elderly that had good quality descriptions and evaluations. Seven evidenced reduced hospital and/or long-term care utilisation and some reported significant savings per case. Key components of these models of care are presented in Appendix 3. The success factors resonate with the report published by the Kings Fund in 2014 [23].

Hoogendijk [24] analysed three different integrated models in the Dutch National Care for the Elderly Program. In the Frail Older Adults Care in Transition (ACT) trial, no significant effect was found on quality of life, psychological health, function, hospitalisation, or costs at 24 months [25]. The Prevention of Care cluster randomised trial reported no significant differences in a range of outcomes. The Utrecht primary care PROactive Frailty Intervention Trial (U-PROFIT) included a multi-component intervention associated with small effects on activities of daily living (ADL)/instrumental activities of daily living (IADL) and dependency but no effects on health-related quality of life, hospitalisations, mortality or satisfaction with care. Looman et al. [26] reported that the Walcheren Integrated Care Model (WICM) had a small effect on health, quality of life, health care use and satisfaction with care after three months. However, in an economic evaluation over 12 months, WICM was not cost-effective as costs per quality-adjusted life year were high [27].

In the French CO-ordination Personnes Agées (COPA) controlled study comparing CGA and intensive case management with usual care, total hospitalisations were unchanged, unplanned admissions declined, and there was no difference in institutionalization or mortality [28]. A quasi-experimental study of case management and multicomponent interventions at home or in a short-term residential setting [29], reported lower institutionalization rates.

A prospective randomised controlled trial [30] of 24-hour support from a Community Geriatrics Unit compared to standard primary care reported a lower hospitalisation rate after the first year, lower first emergency room visits, and patients were more likely to die at home in the intervention group. There was no difference in institutionalization or mortality rates.

### Comprehensive Geriatric Assessment in Hospital

There is strong evidence for the benefits of inpatient CGA delivered by specialist teams in dedicated units [31]. An updated Cochrane review of CGA for adults ≥ 65 years, admitted to hospital as an emergency [16] concluded inpatient CGA was associated with more patients living in their own homes at three to 12 months’ after discharge. A systematic review of CGA for older people assessed, treated and discharged within 72 hours of emergency admission to hospital [32] found only five randomised control trials (RCTs) eligible for analysis. There was no clear evidence of benefit from CGA in terms of mortality, readmissions, institutionalisation, function, quality-of-life or cognition.

### Intermediate Care Services

The report on Better Care for Frail Older People published by the Deloitte Centre for Health Solutions in 2014 [33] recognises the value of investing in intermediate care services that offer safe and effective community based assessment, treatment and rehabilitation alternatives to acute hospital care at times of a deterioration in the health of the older person or their caregiver. Intermediate care is time limited (usually for a period of days or weeks) with a clear objective of prevention of admission and readmission, shortened length of hospital stay, smoother transfer to post-acute care, and reduced need for long term institutional care.

A systematic review of 10 randomised controlled trials of admission avoidance hospital care at home [34] found lower mortality at six months and greater satisfaction for hospital care at home compared to inpatient care. Hospital at home care was less expensive when the analysis was restricted to treatment actually received and when the costs of informal care were excluded. Older patients managed by hospital at home in New Mexico, USA, had comparable or better clinical outcomes and higher satisfaction compared with similar inpatients, achieving 19% reduction in costs [35].

In a home based programme for frail older people with severe and disabling chronic illnesses, access to same day urgent house visits for exacerbations of chronic illness [36] led to 17% lower total Medicare costs compared to matched controls over a mean of two years of follow-up. A quasi-experimental Catalan study of an early supported discharge programme for medical and orthopaedic patients reported that patients receiving Hospital at Home had an average of six days shorter hospital stay and better functional outcomes compared to a propensity matched cohort managed in hospital [37].

In their updated Cochrane review of day hospitals, Brown et al. [38] reported low quality evidence that medical day hospitals appear effective compared to no comprehensive care for the combined outcome of death or poor outcome, and for deterioration in activities of daily living. In a recent scoping review of community hospitals, Pitchforth et al. [39] noted that patient experience was frequently reported to be better at community hospitals, although there was limited evidence for cost-effectiveness.

## IV. DISCUSSION

The literature review identified few models of integrated care specifically designed to prevent and tackle frailty in the community and at the interface between primary care and secondary care. Most were small scale demonstration projects that have yet to scale. This scale up requires a favourable political, funding and organisational context as illustrated by the PAERPA pathway for people at risk of losing their autonomy in France and in Scotland’s Reshaping Care for Older People and Change Fund. Economic benefits of implementing system-level changes at scale are described in the Program of Research to Integrate the Services for the Maintenance of Autonomy (PRISMA) in Quebec [40].

The overview by Béland et al. [22], and the recent empirical studies, illustrate the key components of an effective model of integrated care for frailty: a single-entry point, individualised assessment and care plans, case management, coordination of home and community services across the continuum of care, effective management of care transitions, enabled by an electronic information tool and clear policies and procedures for eligibility and care processes. These components reflect the Multimorbidity Care Model developed by the Joint Action on Chronic Diseases and Promoting Healthy Ageing across the Life Cycle (www.chrodis.eu) and recommendations from the Kings Fund for making our systems fit for an ageing population [23]. They also echo the findings of a recent thematic analysis on factors associated with implementing integrated care for frail older adults [41], and key insights and lessons from a seven-country cross-case analysis of integrated care for older adults and those with complex needs [42].

Based on this evidence and experience, we suggest the key principles for building an effective model of integrated care for frailty are:

### Target frailty

Future models should improve the targeting of interventions towards high-risk frail community-dwelling older adults [43]. This may require a two-step process using a brief frailty-specific screening tool in primary care and community settings, followed by CGA delivered by suitably trained practitioners to identify and target the appropriate frail cohort.

### Promote enablement

Ryburn et al. [17] suggest that a restorative approach has significant advantages over the traditional model of home care maintenance and support. Timely interventions, education and assistive technologies specifically designed to encourage frail older people to resume activity and regain independence may be cost-effective by reducing future demand for services. The frailty prevention approach should incorporate a behavioural health, education and enablement ethos and include interventions that enable the individual to participate in a home exercise programme, regain skills such as cooking or dressing, and build social networks that reduce isolation, depression and anxiety.

### Support self-management

Harrison et al. [6] advocate that a shift from a predominantly biomedical model may be facilitated by framing frailty as a chronic condition and adopting chronic care strategies. An effective holistic approach to frailty would include health education, enablement, rehabilitation and support for the individual to manage their conditions and maintain optimal function, and support for the caregiver to remain well and continue in their caring role.

### Provide continuity and co-ordination of care

Fragmented, reactive and poorly coordinated care for frailty results in poor functional outcomes, creating dependency and further escalating demand and costs [40]. Proactive and coordinated care at home by a continuous partnership between the case manager and family physician is more likely to anticipate events and trigger earlier interdisciplinary interventions to maintain function and delay escalation of dependency. Trusting relationships between care professionals and across the networks of provider organizations are particularly important for managing transitions and anticipating the need for urgent advice and support after hours.

### Tailor multidimensional interventions

For each individual, multiple physical, cognitive, social and functional interventions may be needed to address different dimensions of the frailty syndrome [44]. Selection of interventions should be tailored to the individual’s health conditions, stage of frailty, trajectory of needs, carer support, housing, social circumstances and personal goals. The interventions should be prioritised to avoid risk of overtreatment and adverse events.

### Explore new models of CGA in hospital and in intermediate care alternatives to admission

Ward based specialist led CGA remains the gold standard but where demand exceeds capacity, emerging workforce innovations and shared care models should be evaluated against this evidence based model. Hospital at home alternatives to admission appear to be promising for selected individuals. However, further well-designed trials of CGA for frail older people within more general intermediate care services are required.

### Develop workforce skills and competencies on frailty

Many of the studies established new services that required a long lead time for staff to develop their skills. To be affordable and sustainable, integrated care for frailty must be able to be adopted across the whole community health and care workforce. This will require education and training for frailty in all workforce curricula.

### Support adoption and assure implementation

As adherence to CGA and care planning tends to diminish over time, support for adoption and continuous quality monitoring will be critical to guarantee fidelity and sustain successful implementation. A wide range of technological solutions can enable remote monitoring, self-management, decision support, and electronic sharing of information.

### Improve outcomes for people

Models of care should be designed around outcomes that matter for individuals and their caregivers as well as health and social care systems and provide meaningful societal impact. A focus on patient, client or user-defined goals and outcomes should serve to capture care experience, quality of life and participation outcomes in addition to functional and traditional health and social care metrics.

### Undertake further research and evaluation

Although the methodological approach was rigorous, some relevant studies may not have been captured as the search terms “frail” and “frailty” may have excluded studies of more general models of integrated care for older people or patients with multi-morbidity. To mitigate these issues, we invited representatives of the 22 European Member States participating in the ADVANTAGE consortium to ensure that all relevant studies and grey literatures were included in this systematic review.

As also reported by Briggs et al [10], most studies focused on clinical components of integrated care for frailty with less focus on how to organise and deliver these integrated approaches across the whole pathway and at a system-level.

## V. CONCLUSION

This review concluded that the frailty prevention approach should incorporate key components such as use of simple frailty specific screening tools in all care settings, tailored interventions by interdisciplinary teams in hospitals and community, case management and coordination of support across the continuum of providers, effective management of transitions between care teams and settings, information and technology enabled care solutions, and clarity about service eligibility care policies, procedures and processes. Further research is required to understand how to scale up integrated care for frailty in different systems and how to achieve optimal impact and value.
